# Sleeping habits during COVID-19 induced confinement: A study from Jordan

**DOI:** 10.1016/j.heliyon.2021.e08545

**Published:** 2021-12-03

**Authors:** Mahmoud A. Alomari, Karem H. Alzoubi, Omar F. Khabour, Mohammad Z. Darabseh

**Affiliations:** b Department of Physical Education, Qatar University, Doha, Qatar; a Division of Physical Therapy, Department of Rehabilitation Sciences, Jordan University of Science and Technology, Irbid, Jordan; c Department of Pharmacy Practice and Pharmacotherapeutics, University of Sharjah, Sharjah, United Arab Emirates; d Department of Clinical Pharmacy, Jordan University of Science and Technology, Irbid, Jordan; e Department of Medical Laboratory Sciences, Jordan University of Science and Technology, Irbid, Jordan; f Division of Physiotherapy, Department of Allied Medical Sciences, Aqaba University of Technology, Aqaba, Jordan

**Keywords:** COVID-19, Sleep, Lifestyle, Adults, Jordan

## Abstract

Sleep can significantly modulate the immune response to infectious agents. In the current study, changes in sleep quality during COVID-19-induced confinement among adults were investigated. This was a cross-sectional survey study of the public using social media. Participants (n = 1846) were recruited in the study, of which >92% reported a variety of confinement procedures such as self-quarantine, physical distancing, banning of public events, school closure, and lockdown. Majority of the participants (53–59%) reported an increase in most of the sleep parameters except a decrease (49.1%) in daytime sleep. Age was associated with changes in sleeping disturbances during COVID-19 confinement (*p* < 0.001). Young participants were more likely to experience sleeping disturbance than older ones (*p* < 0.05). In addition, gender (*p* < 0.001) is an independent predictor of nighttime sleeping. Being a male is associated with a “decrease” and being a female is associated with an “increase” in nighttime sleeping hours (*p* < 0.05). Moreover, change in daytime sleeping was related to age, gender, and job type (*p* < 0.05). In conclusion, changes in sleep quality during COVID-19-induced confinement were reported. Intervention programs and strategies are warranted to further improve sleep during the current and future disease-induced confinement.

## 1 Introduction

Coronavirus Disease 2019 (COVID-19) has started in Wuhan, China since December 2019 [1]. Shortly after, the virus has rapidly spread around the globe, compelling the World Health Organization to announce it as a global pandemic [2]. As of 22 October 2021, the number of people infected according to World Health Organization is 242,348,657 and fatality is 4,927,723 around the globe. COVID-19 causes serious damage to the epithelial layers in the respiratory system precipitating an inflammatory process that might lead to acute respiratory distress, and possible death [3, 4]. Furthermore, recent clinical, pharmacological, and laboratory findings confirmed that the virus attacks the central nervous system [5].

COVID-19 can be transmitted in humans via respiratory droplets, contact, fomites, nosocomial and in rare circumstances can be airborne [6]. The transmission of the virus and the high number of cases led governments around the world to impose rules and regulations, including confinement tactics, to help to intercept, or at least, slow down the spread of the virus [7]. These tactics included social distancing, quarantine, shielding, lockdown and complete isolation. Subsequently, people were confined to homes, which affected normal activities, daily routine, lifestyle, and quality of life [8, 9].

Sleep is crucial for sustenance of normal physiological functions [10]. In healthy subjects, lack of sleep contributes to fatigue, depression, and decreased level of physical activity [11]. Sleep is affected by many physical and psychological factors such as pain, quality of life, medication, anxiety, depression, bladder problems and physical activity level [11]. Additionally, sleep quality and mental health can help improve immunity to viral infection [12]. Therefore, mental health and sleep quality are important considerations at the age of COVID-19.

Confinement to homes due to COVID-19 affected several aspects of people's quality of life, including physical activity, mental health [13], social communication, and possibly sleep [14]. Previous studies have shown that the prevalence of novel infectious diseases, such as acute respiratory attacks, increases anxiety, depression, and stress during confinement in the general population [15], subsequently, might disturb sleep [14]. Interestingly, people who live in confined environments such as space and marine missions suffer from sleep impairment and disruption of the circadian rhythms [16, 17], which have been attributed to social isolation, physical inactivity, boredom, monotony and stress [17]. Similarly, people who are confined due to COVID-19 regulations might have disrupted sleep [18]. No studies have confirmed such hypothesis yet in the population of Jordan. Thus, the current study aimed to explore the changes in sleep habits (SH) during COVID-19-induced confinement among adults. The results can help to determine plans and establish strategies to improve sleep hygiene awareness and patterns during the current and similar calamities.

## 2 Method

### 2.1 Design and participants

The current data is derived from a larger cross-sectional study, the “Behavior, Knowledge, Stress and Quality of Life during COVID-19-induced Confinement (BKSQ-COVID-19)”. The study was conducted during the second and third quartiles of 2020. Men and women above 18 years old living in Jordan were included in the study. The participants 18–34 years were considered young adults, 35–49 years were considered middle adults, while above 50 were considered older adults. Individuals with physical, psychological, or mental disorders that prevent them from answering the questions were excluded from the study. The researcher used G∗Power software version 3.1.9.7 to calculate the sample size. A 0.05 significance level, a power of 0.90, and a small effect size of 0.10 required the minimum number of subjects to be 1810. The questionnaire was distributed anonymously and electronically via social media platforms. Before filling the questionnaire, the participants were informed about the study details including study aims, requirements, expected duration, procedures, risks involved, benefits, and rights of refusal/withdrawal. Subsequently, the participants had the choice to agree or refuse to participate. The Institutional Review Board (IRB) approved the study procedures.

### 2.2 Questionnaire

The sleep-related questions included in the instrument were about changes in SH, sleep disturbances, nighttime sleeping, daytime sleeping, and total sleeping hours. The question was: “What changes in the following lifestyle have you experienced during the confinement due to coronavirus? “These lifestyle components included questions about sleeping. These questions were: 1) sleep disturbances, 2) nighttime sleeping, 3) daytime sleeping, and 4) total hours of sleep. The participants selected one of the choices, “increase”, “decrease”, and “no-changes”. The official local language, Arabic, was used in the questionnaire. Additionally, the participants were asked “whether or not were worried to be infected with COVID-19”. The responses were “yes” and “no”.

The questionnaire was pilot tested in 25 participants to ensure quality and comprehensibility. Additionally, subjects from the pilot sample were asked to provide comments about how they understood each survey item to ensure content clarity and comprehension. Pilot samples were omitted from the final analysis. The reliability coefficient for all items of the study was >0.65. As for validity, the study survey was face validated via review by experts in the field, including senior researchers in public health, social sciences, medical sciences, and research ethics.

### 2.3 Statistics

The data was entered and coded in the SPSS (version 21) for statistical analysis and presented as mean ± SD, frequency and percentages. Significance (*p*-value) was set at 0.05. To examine the differences in the participant responses to the sleeping questions, the ꭓ^2^ goodness-of-fit was used. Additionally, multinomial logistic regression was used to determine the relationship of potential factors with the participant responses to the questions. The potential factors were age, gender, obesity, and education level [19]. The responses to the questions that used for all statistical analysis were “increase”, “decrease”, and “no-changes”.

## 3 Results

### 3.1 Participants

Since the survey was web-based, it is difficult to estimate the response rate to the invitation to participate the study. A total of 1846 participants were agreed to participate in the study, among which 89 (4.1%) provided no sleeping data, thus excluded from the analysis. Table 1 shows average age (Median = 32.00; IQR = 17.0; range: 18–72 years), weight (Median = 70.00; IQR = 23.0; range: 38–144 kg), and height (Median = 165; IQR = 13.0; range: 120–198 cm). The majority of the participants were women (69.4%), from a middle-income class (77.0%) while 51.6% held a bachelor's degree and 35.6% were unemployed. Table 2 shows the confinement procedures with about 40.4% indicated being worried about getting infected with COVID-19.

**Table 1:** The participant demographic (n = 1757).

Gender (%; men)	30.6
Age (yrs, mean ± SD)	33.8 ± 11.1
Weight (kg, mean ± SD)	72.8 ± 16.2
Height (cm, mean ± SD)	166.3 ± 9.8
Level of Education (%)	
High school and less	19.0
Associate degree	13.9
Bachelor degree	51.6
Graduate degree	15.5
Income (%)	
Low	15.5
Middle	77.0
High	7.5
Job type (%)	
Unemployed/retired	35.6
Military/Police	4.8
Education	23.7
Agriculture	1.7
Health	14.1
Manufacturing	2.9
Engineering	5.7
Management	8.3
Crafting	3.2

**Table 2:** Confinement information due to COVID-19 (n = 1757).

Likelihood of getting infected	(%)
Low	59.6
High	40.4
Self-quarantine	
Yes	93.6
No	6.4
Physical distancing	
Yes	96.8
No	3.2
Banning group events (i.e. weddings)	
Yes	98.2
No	1.8
School closure	
Yes	99.0
No	1.0
Lockdown	
Yes	96.9
No	3.1

### 3.2 Changes in sleeping behavior

The goodness-of-fit chi-square test revealed differences (*p* < 0.0001) in the responses to the SH questions, “increase”, “decrease”, versus “no-change”. Table 3 shows that >50% (range: 53.1%–59.4%) of the participants reported an “increase” in sleep disturbance, nighttime sleeping, and total sleeping hours while 49.1% reported a “decrease” in daytime sleeping.

**Table 3:** Prevalence of changes in sleeping habits (n = 1846).

Sleeping habits	Decreased	No change	Increased	ꭓ^2^; *p*-value
Sleeping disturbance (%)	12.1	28.5	59.4	562.0; 0001
Night sleeping hours (%)	18.3	28.6	53.1	297.9; 0001
Daytime sleeping hours (%)	49.1	25.7	25.3	195.6; 0001
Total sleeping hours (%)	15.7	28.2	56.1	443.4; 0001

Values presented are the percent (%) of participants.

### 3.3 Factors contributing to the changes in sleeping habits

Table 4 demonstrate the regression for the contributing factors to changes in sleeping habits. The multinomial regression showed that the overall statistical model can predict (ꭓ^2^ = 40.0; *p* = 0.0001) changes in sleeping disturbances during COVID-19 confinement. Further analysis revealed that only age (ꭓ^2^ = 20.2; *p* = 0.0001) was associated with changes in sleeping disturbances during COVID-19 confinement. Younger age (*β* = -0.02; OR = 0.98; *p* = 0.05) was associated with an “increase” in sleeping disturbance while older age (*β* = 0.02; OR = 1.0; *p* = 0.04) was associated with a “decrease” in sleeping disturbance. No differences in all sleeping indices (total sleeping hours ꭓ^2^ = 1.4; sleep disturbances: *p* = 0.4, daytime sleeping: ꭓ^2^ = 0.5; *p* = 0.7, nighttime sleeping: ꭓ^2^ = 1.5; *p* = 0.4, ꭓ^2^ = 5.4; *p* = 0.065) were found between the ones who were “worried” versus the ones “not-worried” to be infected.

**Table 4:** Regression of contributing factors to changes in sleep habits during COVID-19.

	Total hours of sleep		Sleeping disturbance		Nighttime Sleeping hours		Daytime Sleeping hours	
	Exp(B)	95%CI	Exp(B)	95%CI	Exp(B)	95%CI	Exp(B)	95%CI
Decrease								
Age	0.956	0.94–0.97‡	0.984	0.97–1.00‡	0.991	0.98–1.00	0.957	0.94–0.97‡
Obesity	1.000	0.97–1.03	1.005	0.97–1.04	0.979	0.953–1.01	0.993	0.96–1.02
Gender (Women)∗	1.88	1.4–2.6‡	1.2	0.84–1.7	1.4	1.1–1.87‡	1.4	1.03–1.93‡
Education Degree								
High school†	0.713	0.43–1.17	0.471	0.27–0.82	0.924	0.62–1.38	0.493	0.30–0.82‡
Associated†	0.802	0.470–1.37	0.881	0.46–1.67	0.891	0.57–1.40	0.508	.30-0.87‡
Bachelor†	0.277	0.511–1.21	0.780	0.47–1.31	1.045	0.74–1.47	0.631	0.40–1.01‡
Increase								
Age	0.97	0.93–1.01	1.022	1.00–1.04‡	0.988	0.95–1.03	1.009	0.99–1.03
Obesity	1.019	0.93–1.11	0.968	0.92–1.017	1.004	0.916–1.10	0.990	0.95–1.03
Gender (Women)∗	1.88	0.6–5.2	1.5	0.96–2.55	3.3	1.4–7.9‡	0.48	0.30–0.6‡
Education Degree								
High school†	2.010	0.39–10.38	0.377	0.181–0.79	3.083	0.34–28.20	0.383	0.20–0.75‡
Associated†	2.331	0.42–12.90	0.654	0.29–1.50	9.085	10.9–75.69‡	0.437	0.22–0.87‡
Bachelor†	0.584	0.11–3.19	0.570	0.3–1.09	3.763	0.47–29.90	0.666	0.38–1.18

‡ *p* < 0.05.

∗ Versus men.

† Versus Graduate degree.

According to another multinomial regression, the model can predict (ꭓ^2^ = 26.7; *p* = 0.008) changes in nighttime sleeping during COVID-19 confinement. Additional analysis showed that only gender (ꭓ^2^ = 13.4; *p* = 0.001) is an independent predictor of nighttime sleeping. Being a male is associated with a “decrease” (*β* = 0.36; OR = 1.4; *p* = 0.006) and being a female is associated with an “increase” (*β* = 2.2; OR = 9.1; *p* = 0.04) in nighttime sleeping hours.

Additional multinomial regression revealed that the model can predict (ꭓ^2^ = 108.1; *p* = 0.0001) the changes in daytime sleeping hours during COVID-19 confinement. Analysis indicated that daytime sleeping hours were associated with age (ꭓ^2^ = 62.8; *p* = 0.0001), gender (ꭓ^2^ = 31.4; *p* = 0.0001), and education (ꭓ^2^ = 13.5; *p* = 0.004). A reduction in age (*β* = -0.043; OR = 0.96; *p* = 0.0001), being a male (*β* = 0.34; OR = 1.41; *p* = 0.03) and having a high school (*β* = -0.70 OR = 0.50; *p* = 0.007), two-year (*β* = -0.67; OR = 0.50; *p* = 0.01), and four-year (*β* = -0.46; OR = 0.63; *p* = 0.05) diplomas are associated with a “decrease” in daytime sleeping hours. Additionally, being a male (*β* = -0.73; OR = 0.5; *p* = 0.002) and having a high school diploma (*β* = -1.0 OR = 0.4; *p* = 0.005) and a two-year degree (*β* = -0.83; OR = 0.43; *p* = 0.02) were associated with an “increase” in daytime sleeping hours.

The multinomial logistic regression also showed that the overall statistical model can predict (ꭓ^2^ = 67.1; *p* = 0.0001) changes in sleeping hours during COVID-19 confinement. However, additional analysis revealed that only age (ꭓ^2^ = 44.1; *p* = 0.0001) and gender (ꭓ^2^ = 15.2; *p* = 0.001) were related to changes in total sleeping hours. A reduction in age (*β* = -0.045; OR = 0.95; *p* = 0.0001) and being a male (*β* = 0.64; OR = 1.90; *p* = 0.0001) are associated with an “increase” in sleeping hours.

## 4 Discussion

COVID-19 has caught the world unprepared precipitating drastic changes in lifestyle, including compulsory confinement [20]. Theoretical studies have warranted of the possible health ramifications of COVID-19-induced confinement, particularly sleep disturbances [21]. The current study examined the changes in SH among adults. The results indicate that the majority (53.1%–59.4%) of the participants reported an increase in most of the sleep parameters during COVID-19 pandemic except a decrease (49.1% of the participants) in daytime sleep. Additionally, age, gender and level of education seemed to contribute to these changes in SH. The current study is a novel study that examined the changes in SH among adults during COVID-19-induced confinement. The study findings are of importance to address sleep changes and identify the factors that might affect sleep quality while in quarantine. Subsequently, prepare plans and implement strategies to improve sleep quality during calamity-induced confinement, particularly due to infectious diseases. Especially, the pandemic is wildly spreading and its end is not foreseeable, as to yet.

Compulsory confinement is usually experienced under exceptional circumstances including imprisonment, space trips, and Antarctic camps [16, 17]. The current results confirm previous findings suggesting altered SH during confinement. These studies suggested that people might have long hours of sitting in the same place without conducting physical activity, which might be associated with boredom and stress [17]. Subsequently, human psychology and physiology are significantly affected leading to sleep alteration [22]. For example, Chu et al. (2015) found that some people had improvement in sleep while other participants reported lower sleep quality after nighttime confinement in patients with mental disorders [23]. Interestingly, Hocking (1970) attributed the increase in nightmares to depression, anxiety, phobias, confusion, and impaired memory and concentration after living in isolated environments [24]. Similarly, it has been reported that incarcerated women suffer from poor sleep due to altered mental and psychological health including anxiety, depression, and traumatic memories [25]. Consistent with these findings, increased depression due to poor sleep quality was associated with substance (i.e. drugs, cigarettes, and caffeine) abuse, and crime rate (i.e. rape and assault) in prisoners [26]. All these findings suggest the importance of sleep quality for overall health.

In the current study, most of the participants reported a decrease in daytime sleeping while many experienced an increase in nighttime sleeping and total sleeping time. This is probably healthy SHs as most recommendations emphasize avoiding napping, especially during the daytime, to improve sleep quality. Avoiding napping is important for consolidating nighttime sleep for optimal sleeping hygiene. These changes might be due to participants were busy following the COVID-19 news during the daytime, especially during the early stage of the pandemic. These results suggest people avoided daytime sleeping to consolidate nighttime sleeping hours. Conversely, sleeping disturbance increased among ∼50% of the participants, which is a sign of unhealthy SHs. This increase might be due to the stress that the participants might be experiencing due to the confinement and the spread of COVID-19 pandemic [19]. However, these findings and speculations need further confirmations in future studies.

Sleep, in general, is affected by many physical and psychological factors such as age, gender, physical activity level, life circumstances, medication, pain, bladder problems, anxiety, and depression [11]. The current study confirms the importance of demographics (i.e. age and gender) and socioeconomic status (i.e. education) for sleep quality. Friedman et al. (2007) found that socioeconomic status is highly correlated with sleep quality in aging women. It was suggested that the links between socioeconomic status, including income and education level, with sleep quality are explained in part by psychosocial measures, specifically depression [27]. Depression, which is associated with lower socioeconomic levels, may be associated with lower sleep quality [27]. The contribution of age, predominantly older adults, and gender, mainly women have higher sleep impairments than men, to sleep disturbance can be attributed to the changes in hormonal levels between men and women and with changes in age [28]. This is explained by the age- and gender differences in sleep circadian hormone, specifically: melatonin, cortisol and serotonin [29].

### 4.1 Implications

Current findings identified changes SH and relating factors while in pandemic-induced quarantine. The participants reported some healthy SHs including a decrease in daytime sleeping and an increase in nighttime and total sleeping, which should be encouraged. The results are important to characterize specific groups that are at risk of developing sleep impairments. Subsequently, provide proper recommendations and advice for sleep management during confinement due to COVID-19 and future pandemics. These recommendations can include participation in physical activity, frequent interaction with family members within the same household, regular night-time sleep and wake-up time schedule, stress management and creating a quiet and attractive environment suitable for sleep [21]. Future studies are needed to confirm the current findings and verify these speculations.

### 4.2 Limitations

The study was conducted using Google forms and thus the response rate cannot be calculated. The study aimed at knowing the changes in sleeping habits, thus the exact time of sleeping parameters was not collected. Therefore, it is recommended in future investigations to examine the total sleeping time and changes before and after the pandemic.

## 5 Conclusions

The current study examined changes in sleep quality during COVID-19 -induced confinement. The majority of the participants experienced an increase in sleep disturbance, nighttime sleeping, sleeping hours as well as a reduction in daytime sleeping. Furthermore, the data showed that age, gender, income, and job type seemed to contribute to these changes. However, studies and interventions are needed to confirm and understand the current results. Yet, programs and strategies are warranted to further improve sleep among adults during the current and future disease-induced confinement.

## Declarations

### Author contribution statement

Mahmoud A. Alomari, Karem H. Alzoubi and Omar F. Khabour: Conceived and designed the experiments; Performed the experiments; Analyzed and interpreted the data; Wrote the paper.

Mohammad Z. Darabseh: Conceived and designed the experiments; Analyzed and interpreted the data; Wrote the paper.

### Funding statement

This work was supported by via Deanship of Research, 10.13039/501100004035Jordan University of Science and Technology (grant number 245/2020). The publication of this article was funded by the Qatar National Library.

### Data availability statement

Data will be made available on request.

### Declaration of interests statement

The authors declare no conflict of interest.

### Additional information

No additional information is available for this paper.
